# The mode of progressive disease affects the prognosis of patients with metastatic breast cancer

**DOI:** 10.1186/s12957-018-1472-9

**Published:** 2018-08-14

**Authors:** Ryutaro Mori, Manabu Futamura, Kasumi Morimitsu, Yoshimi Asano, Yoshihisa Tokumaru, Mai Kitazawa, Kazuhiro Yoshida

**Affiliations:** Department of Surgical Oncology, Gifu University Graduate School of Medicine, 1-1 Yanagido, Gifu, 501-1194 Japan

**Keywords:** Secondary breast neoplasms, Drug therapy, Progressive disease, Prognosis

## Abstract

**Background:**

According to the Response Evaluation Criteria in Solid Tumors (RECIST), progressive disease (PD) is diagnosed under two conditions: an increase in size of pre-existing lesions (IS) and the appearance of new lesions (NL). We retrospectively investigated the difference in the prognosis between IS and NL.

**Methods:**

Patients receiving drug therapies for metastatic breast cancer between 2004 and 2015 at our institution were reviewed. The survival time after NL and IS was compared and the frequency of NL with each drug calculated.

**Results:**

For the 107 eligible patients, the survival time after NL at second-line chemotherapy was significantly worse than after IS (median survival time 4.3 months vs. 20.3 months, *p* = 0.0048). Maintenance therapy with bevacizumab or trastuzumab had a high frequency of NL (88.9%), and third-line eribulin had a low frequency of NL (16.7%). A multivariate analysis showed that NL at second-line chemotherapy was not an independent risk factor (hazard ratio 1.02, 95%; confidence interval 0.54–1.93, *p* = 0.95) for the total survival time.

**Conclusions:**

Patients with IS had a better survival after PD than those with NL. We may be able to avoid changing drug therapy for patients without NL and allow them to continue drug therapy for longer.

## Background

Patients with metastatic breast cancer (MBC) are treated with drug therapies, such as hormone therapy and chemotherapy. Although MBC is still an incurable disease [1], the survival of such patients has been improved by new therapeutic agents [2, 3]. The efficacy of drug therapies is evaluated by imaging modalities, such as computed tomography (CT), bone scintigraphy, and positron emission tomography (PET), and the therapy is continued as long as it seems to be effective [4]. The efficacy of drug therapies is evaluated based on Response Evaluation Criteria in Solid Tumors (RECIST) especially in clinical trials [5] as well as in daily practice.

Under the RECIST criteria, the efficacy is divided into four categories: complete response (CR), partial response (PR), stable disease (SD), and progressive disease (PD). Usually, the therapy is changed when the efficacy is evaluated as PD. According to the RECIST criteria, PD is diagnosed under two conditions: an increase in the size of pre-existing lesions and the appearance of new lesions [5]. These situations should be interpreted as indicative of the progress of metastatic disease. However, the precise meanings of these situations seem to differ, as the size of pre-existing lesions may be decreased again by subsequent therapy, while new lesions rarely disappear, regardless of therapy. Thus, the European Medicines Agency (EMA) has recently recommended that the mode of progressive disease (an increase in the size of pre-existing lesions or the appearance of new lesions) should be taken into account when progression-free survival (PFS) is used as the endpoint of a clinical trial [6].

Given the above, we suspected that the prognosis of patients with new metastatic lesions might differ from that of patients whose pre-existing metastatic lesions have only increased in size. Therefore, we retrospectively investigated the prognosis of these patients after PD and the relationship between the mode of PD and drugs.

## Methods

The records of breast cancer patients who received drug therapy for locally advanced or metastatic breast cancer at Gifu University Hospital between 2004 and 2015 were reviewed. The therapy in each case was investigated, and the efficacy of drug therapy was evaluated from the viewpoint of the objective response, which was categorized as mentioned above: CR, PR, SD, and PD. This categorization was determined based on the RECIST criteria.

In the present study, PD was further divided into the appearance of new lesions (NL) and an increase in the size of existing lesions (IS). NL indicates that the patient developed new metastatic lesions that had never been detected before, regardless of the size of pre-existing lesions. IS indicates that the target lesions had increased in size, and no new lesions had developed. When judging the mode of PD, the most recent therapies the patients had received were excluded, as the efficacies of these therapies were deemed poor and had not been evaluated sufficiently in most cases. We then compared the survival after PD between the patients with NL and IS using the Kaplan–Meier curves with a log-rank test and calculated the frequency of NL to determine which drugs was most strongly associated with NL. We also analyzed the impact of these factors on the patients’ survival using a Cox proportional hazard model. All statistical analyses were carried out using the software EZR software program (version 3.4.1 with R commander 2.4–0).

This study was approved by the ethics committee of Gifu University, Graduate School of Medicine.

## Results

### Patient characteristics

A total of 127 patients received drug therapy for locally advanced or metastatic breast cancer in our institution. However, 20 patients were excluded because their outcomes were unclear. Thus, we investigated the outcomes of the 107 remaining patients. The patients were a median 58 years of age. Most of the primary tumors exhibited a size of 2–5 cm (T2). Seventy-four patients had *N* (+) status, 74 had estrogen-receptor (ER)-positive tumors, and 22 had HER2-positive tumors. The metastatic sites included the bones (61 patients), lungs (34 patients), liver (29 patients), lymph nodes (44 patients), pleural (26 patients), local (15 patients), and other organs (17 patients). The details are shown in Table 1.

**Table 1:** Patient characteristics

*n* = 107	No
Age, years	
< 50	20
50–59	38
≥ 60	49
Menopausal status	
Premenopausal	22
Postmenopausal	85
T factor	
T1	15
T2	43
T3	7
T4	20
Unknown	22
Nodal status	
*N* (−)	19
*N* (+)	74
Unknown	14
Subtypes	
Luminal A/B	68
Luminal HER2	8
HER2	14
Triple negative	17
Metastatic sites	
Bone	61
Lung	34
Liver	29
Lymph node	44
Pleural	26
Local	15
Brain	9
Others	8

### Selection of drug therapy

The patients received a median of four lines of drug therapy. Among those who received chemotherapies, oral 5-FU derivatives were administered to 66 cases, taxanes (excluding paclitaxel + bevacizumab therapy) to 47 cases, and anthracyclines to 17 cases. Patients with HER2-positive status also received trastuzumab (33 cases) or trastuzumab + pertuzumab (4 cases), and paclitaxel + bevacizumab was administered to 11 cases. Nine of the cases who had been receiving cytotoxic drugs with targeted drugs, such as trastuzumab, pertuzumab, and bevacizumab, received targeted drugs alone after metastatic lesions were well-controlled. Among the cases who received hormone therapies, non-steroidal aromatase inhibitors (AIs) were administered to 66 cases, steroidal AIs to 36 cases, and selective estrogen receptor modulators (SERMs) to 52 cases. The details are shown in Table 2.

**Table 2:** Selection of hormone therapy and chemotherapy

	No.
Chemotherapy	
Oral 5FU	62
Taxane	47
Anthracycline	17
Vinorelbine	17
Eribulin	12
Paclitaxel + Bevacizumab	11
Targeted drug alone	9
Others	26
Hormone therapy	
Non-steroidal AI	66
Steroidal AI	36
SERM	52
SERD	9
MPA	6

*AI* aromatase inhibitor, *SERM* selective estrogen receptor modulator, *SERD* selective estrogen receptor down-regulator, *MPA* medroxyprogesterone 17-Acetate

### Comparing the survival time after NL or IS

**Fig. 1:**
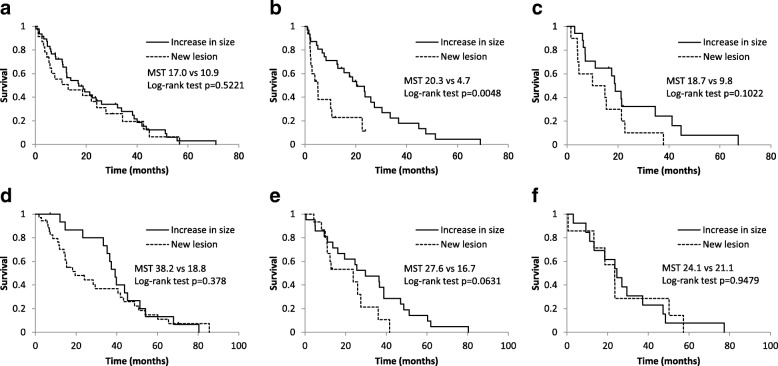
Kaplan–Meier curves for patients with an increase in the size of existing lesions (IS) and the appearance of new lesions (NL). The survivals after PD at (**a**) first-line chemotherapy, (**b**) second-line chemotherapy, (**c**) third-line chemotherapy, (**d**) first-line hormone therapy, (**e**) second-line hormone therapy, and (**f**) third-line hormone therapy

Among the cases who received chemotherapy, the survival time of the patients with NL tended to be worse than that with IS (Fig. 1a–c). After second-line chemotherapy, in particular, the patients with NL had a shorter survival time than did those with IS (median survival time 4.3 months vs. 20.3 months, *p* = 0.0048) (Fig. 1b). Similarly, among the cases treated with hormone therapy, the survival time of the patients with NL tended to be worse than that of those with IS, albeit not to a statistically significant degree (Fig. 1d–f).

### Frequency of NL by drugs

As mentioned above, the patients with NL tended to have poor prognosis after PD. Therefore, we analyzed the frequency of NL for each drug.

**Fig. 2:**
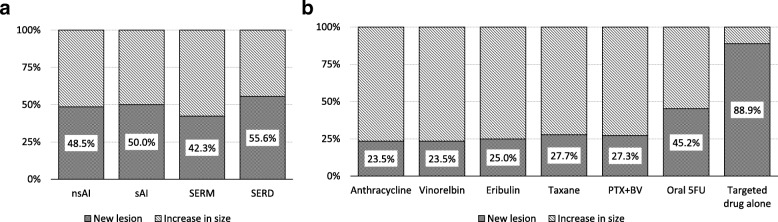
The frequency of new lesions (NL) in hormone therapy and chemotherapy. (**a**) The frequency of NL in the drugs used for hormone therapy. (**b**) The frequency of NL in the drugs used for chemotherapy and targeted drugs (bevacizumab, trastuzumab or trastuzumab combined with pertuzumab). nsAI, non-steroidal aromatase inhibitor; sAI, steroidal aromatase inhibitor; SERM, selective estrogen receptor modulator; SERD, selective estrogen receptor down-regulator; PTX + BV, paclitaxel with bevacizumab

All of the drugs used for hormone therapy had similar frequencies of NL (nsAI 48.5%, sAI 50.0%, SERM 42.3%, and SERD 55.6%) (Fig. 2a). The patients treated with oral s and maintenance therapy with targeted drugs alone (such as bevacizumab and trastuzumab) frequently developed NL (oral 5FUs 45.2% and targeted drug alone 88.9%). Anthracycline, vinorelbin, eribulin, taxanes, and paclitaxel with bevacizumab had similar frequencies of NL (Fig. 2b).

**Fig. 3:**
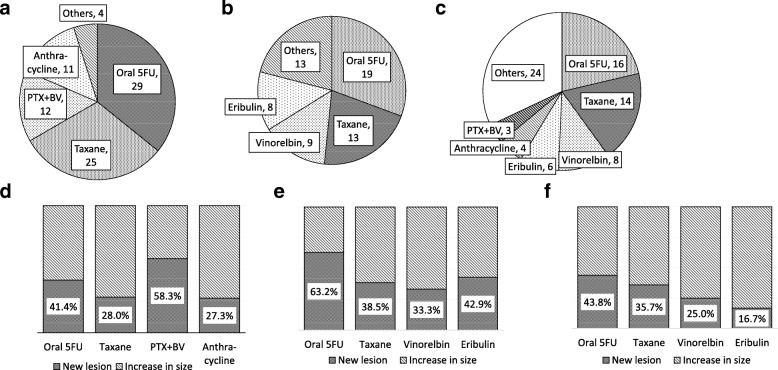
The distribution of the chemotherapy drugs used in first-, second-, and third-line chemotherapy and the frequency of new lesions (NL) in each drug. The distribution of the chemotherapy drugs at (**a**) first-line chemotherapy, (**b**) second-line chemotherapy, and (**c**) third-line chemotherapy, and the frequency of NL at (**d**) first-line chemotherapy, (**e**) second-line chemotherapy, and (**f**) third-line chemotherapy. PTX + BV, paclitaxel with bevacizumab

We also calculated the frequency of NL among chemotherapy drugs stratified by treatment lines. Paclitaxel with bevacizumab therapy had the highest frequency of NL (58.3%) among first-line therapies (Fig. 3a), and oral 5FUs had the second-highest frequency of NL (41.4%) among first-line therapies and the highest (63.2%) among second-line therapies (Fig. 3b). Eribulin had the lowest frequency of NL (16.7%), even among third-line therapies (Fig. 3c).

### A multivariate analysis for the survival after PD with second-line chemotherapy

We analyzed the impact of the factors that seemed to be important for the survival (NL at second-line chemotherapy, targeted therapy alone, eribulin administration, ER-positive status, visceral metastasis at first recurrence) on the survival time after the first recurrence using a Cox proportional hazard model.

As shown in Table 3, NL at second-line chemotherapy was not an independent factor for the survival after the first recurrence (hazard ratio [HR] 1.02, 95% confidence interval [CI] 0.54–1.93, *p* = 0.95), although it was related to a poor survival after PD with second-line chemotherapy, as described above. The administration of maintenance therapy with targeted therapy alone was an independent factor for a good prognosis (HR 0.27, 95% CI 0.12–0.62, *p* < 0.01), although it was related to a high frequency of NL, as described above. The administration of eribulin was also an independent factor for a good prognosis (HR 0.49, 95% CI 0.24–0.99, *p* < 0.05).

**Table 3:** A multivariate analysis for survival after the first recurrence

Factors	Hazard ratio	95% CI	*p* value
New lesions at second chemotherapy	1.02	0.54–1.93	0.95
ER (+)	0.28	0.16–0.48	< 0.01
Targeted therapy alone	0.27	0.12–0.62	< 0.01
Eribulin administration	0.49	0.24–0.99	< 0.05
Visceral metastasis at first recurrence	1.10	0.70–1.73	0.68

ER: estrogen receptor

## Discussion

We analyzed the relationship between the mode of PD and the prognosis and found that the survival time in patients who developed NL at second-line chemotherapy was statistically worse than that in patients with IS.

Some reports have described the relationship between the mode of PD and the prognosis. In the RECORD-1 study investigating the benefit of everolimus over placebo for metastatic renal cell carcinoma, a multivariate Cox proportional hazard model revealed that the growth of non-target lesions and appearance of new lesions were predictive factors for the overall survival [7]. In the Nordic VI trial comparing FLIRI therapy and Lv5FU2-IR therapy for metastatic colorectal cancer, patients with new lesions or unequivocal progression of non-measurable lesions had a worse prognosis than those with an increase in the size of pre-existing lesions, and a ≥ 10% decrease in the size of pre-existing lesions was a positive prognostic factor [8]. Another report has also described the influence of the mode of PD on the overall survival in MBC. Twelves et al. reported that, in study 301 and study 305, which investigated the efficacy of eribulin for MBC, patients who developed new metastases had a worse prognosis than did those with PD due to the growth of pre-existing lesions [9]. Kotake et al. also reported similar findings based on the data from Japanese patients receiving eribulin [10]. The results of these studies have consistently suggested that new lesions are associated with a worse prognosis than IS after PD, which agrees with the findings of the present study. These present and previous findings suggest that simply observing MBC patients whose lesions have slightly increased in size without changing the therapy may be feasible, as long as new lesions do not appear.

Litiere et al. also reported that an increase in the size of target lesions had a low explanatory value, and the appearance of new lesions and the progression of non-target lesions resulted in a worse overall survival, according to the results of a meta-analysis investigating the explanatory values of the mode of PD based on the RECIST criteria in breast, lung, and colorectal cancer. Therefore, those authors suggested that this information be included in the updated version of RECIST [11]. According to the current consensus, the drug therapy in MBC patients must be changed when PD develops [1]. However, we have limited effective drugs. Therefore, if we were to distinguish the treatment protocol after PD between patients with NL and those with IS, we could continue otherwise effective drug therapies for longer.

Another important finding of our study is that NL at second-line chemotherapy itself was not an independent factor for the total survival. NL at second-line chemotherapy is related to a poor prognosis after PD, and patients receiving maintenance therapy frequently develop NL. However, this does not necessarily mean that the total survival time is shortened. The mode of PD was a factor for predicting not the total survival time but only the survival time after PD.

Despite the small population and retrospective nature of the present study, we believe that our results provide some important evidence to support our hypothesis and that these findings may help prolong the survival of patients using limited drugs for metastatic breast cancer. A larger prospective study investigating the prognoses of patients with NL and IS will be required in the future.

## Conclusion

We found that patients with NL had a worse survival after PD than did those with IS. When patients develop NL, their therapy must be changed. However, patients with only IS might be able to be observed without changing their therapy, allowing them to continue otherwise effective drug therapies for longer.

## Abbreviations

AI: Aromatase inhibitor
CR: Complete response
CT: Computed tomography
ER: Estrogen receptor
HER2: Human epidermal growth factor receptor 2
IS: Increase in size of pre-existing lesions
MBC: Metastatic breast cancer
MST: Median survival time
NL: Appearance of new lesions
PD: Progressive disease
PET: Positron emission tomography
PR: Partial response
PR: Progesterone receptor
RECIST: Response Evaluation Criteria in Solid Tumors
SD: Stable disease
SERD: Selective estrogen receptor degrader
SERM: Selective estrogen receptor modulator
